# The *Chlamydia psittaci* Inclusion Membrane Protein 0556 Inhibits Human Neutrophils Apoptosis Through PI3K/AKT and NF-κB Signaling Pathways

**DOI:** 10.3389/fimmu.2021.694573

**Published:** 2021-08-13

**Authors:** Zhangping He, Jian Xiao, Jianye Wang, Simin Lu, Kang Zheng, Maoying Yu, Jie Liu, Chuan Wang, Nan Ding, Mingxing Liang, Yimou Wu

**Affiliations:** ^1^Institute of Pathogenic Biology, Hengyang Medical College, University of South China, Hengyang, China; ^2^Hunan Province Cooperative Innovation Center for Molecular Target New Drug Study, University of South China, Hengyang, China; ^3^Hunan Provincial Key Laboratory for Special Pathogens Prevention and Control, University of South China, Hengyang, China; ^4^Department of Clinical Laboratory, The Affiliated Nanhua Hospital of University of South China, Hengyang, China; ^5^Department of Clinical Laboratory, The Affiliated Huaihua Hospital of University of South China, Huaihua, China

**Keywords:** *Chlamydia psittaci*, CPSIT_0556, hPMN, apoptosis, PI3K/AKT pathway, NF-κB pathway

## Abstract

Inclusion membrane proteins (Incs) play an important role in the structure and stability of chlamydial inclusion and the interaction between *Chlamydia* spp. and their hosts. Following Chlamydia infection through the respiratory tract, human polymorphonuclear neutrophils (hPMN) not only act as the primary immune cells reaching the lungs, but also serve as reservoir for Chlamydia. We have previously identified a *Chlamydia psittaci* hypothetical protein, CPSIT_0556, as a medium expressed inclusion membrane protein. However, the role of inclusion membrane protein, CPSIT_0556 in regulating hPMN functions remains unknown. In the present study, we found that CPSIT_0556 could not only inhibit hPMN apoptosis through the PI3K/Akt and NF-κB signaling pathways by releasing IL-8, but also delays procaspase-3 processing and inhibits caspase-3 activity in hPMN. Up-regulating the expression of anti-apoptotic protein Mcl-1 and down-regulating the expression of pro-apoptotic protein Bax could also inhibit the translocalization of Bax in the cytoplasm into the mitochondria, as well as induce the transfer of p65 NF-κB from the cytoplasm to the nucleus. Overall, our findings demonstrate that CPSIT_0556 could inhibit hPMN apoptosis through PI3K/Akt and NF-κB pathways and provide new insights towards understanding a better understanding of the molecular pathogenesis and immune escape mechanisms of *C. psittaci*.

## Introduction

*C. psittaci* is a zoonotic pathogen that can cause respiratory and digestive diseases in birds, as well as severe pneumonia and bacteremia in humans. Transmission occurs through the respiratory tract to other tissues, and may present as a severe systemic infection, which ranges from mild to severe (1). The developmental cycle of Chlamydia is divided into an infectious elementary body (EB) and a reticulate body (RB). The entry of extracellular infectious EB into host cells is most likely initiated by the induction of endocytosis (2). Upon entry into host cells, the EB differentiates into a non-infectious but metabolically active reticulum (RB). After multiple replications, the progeny RBs differentiate again into EBs and spread to neighboring cells, restarting the infectious cycle (3). EB and RB are membrane-bound and form various types of inclusion bodies in host cells (4). Some proteins are expressed and inserted into the inclusion membrane to modify the inclusion membrane to escape the host endocytosis pathway and survive stably in the host cell, these proteins are termed Incs (5). Our group identified CPSIT_0556 as a metaphase expressed protein, located in the inclusion membrane, and has the same structural characteristics as CPSIT_0844 and CPSIT_0846 (6).

Human polymorphonuclear neutrophils (hPMN) are the first line of defense in the early stages of infection (7) and have a short lifespan. *In vitro* they play a key role in natural antimicrobial defense and are characterized by constitutive apoptosis (8). The complex role of neutrophils in severe pneumonia highlights the activity of specific molecules and processes that promote lung immunity but can also contribute to the progression of severe disease (9). Some microorganisms use hPMN as a Trojan horse and then infect other cells, leaping over various barriers to reach various parts of the body, such as *Leishmania* (10, 11), *C. pneumoniae* (12), and *Klebsiella pneumoniae* (13). Previous studies have demonstrated the intracellular survival of *C. trachomatis* and *C. psittaci* in hPMN (14). hPMN have subsequently been confirmed to be the host cells of *C. pneumoniae* (15). During replication, *Chlamydia* spp. regulate apoptosis using host cell mechanisms, as part of the intracellular survival strategy. *Chlamydia* spp. initially inhibit apoptosis, thus providing ecological niches for their parasitism, while in the mid-to-later stages of replication, *Chlamydia* spp. induce cell apoptosis to enable propagation (16). Incs are involved in the establishment of intracellular replication niches, enabling the survival and efficient proliferation of Chlamydia in host cells (17, 18). However, whether the Incs can inhibit hPMN apoptosis and the mechanisms involved remain unclear. The inhibition of hPMN apoptosis by Incs may be associated with the mechanism of *C. psittaci* immune escape.

Phosphatidylinositol 3-kinase (PI3K) is an intracellular enzyme with serine/threonine kinase activity, which can activate AKT kinase, an anti-apoptotic molecule, and thereby, directly controls apoptosis by regulating the members of the apoptosis cascade (19, 20). The PI3K/Akt signaling pathway is an important survival signaling pathways in hPMN (21). It has been reported that spontaneous apoptosis of hPMN is delayed after activation of NF-κB (22). When the NF-κB pathway is inhibited, the apoptosis of hPMN is induced (23). Furthermore, the p38/MAPK pathways also regulate the apoptosis of hPMN (24). However, there is no data confirming that these signaling pathways are involved in CPSIT_0556-stimulation of hPMN.

Myeloid leukemia 1 (Mcl-1) promotes hPMN survival by binding and isolating Bak and Bax, which can form pores in the mitochondrial membrane (25). While Mcl-1 protein levels gradually decrease during hPMN death (26), its expression can be up-regulated by anti-apoptotic factors, such as PI3K and NF-κB, at the transcriptional and translational levels (27). Conversely, Bcl-2 associated X protein (Bax) is an essential protein involved in apoptosis induction, and its translocation and redistribution to the mitochondria are essential for the implementation of the apoptotic pathway (28). Thus, Bax is considered a quantitative marker of early apoptotic events (29, 30). Anti-apoptotic stimulation inhibits the insertion of Bax into the mitochondrial membrane, thereby inhibiting its pro-apoptotic activity (31).

After pathogen infection, hPMN secrete IL-8, a cytokine which is not only closely related to respiratory diseases (32), but can also be used as a therapeutic target for inflammatory diseases (33). IL-8 has been detected in the supernatant of hPMN infected with *C. pneumoniae* and has been reported to play a role in inhibiting the apoptosis of hPMN (15). It has been shown that IL-8 potentially contributes to mechanisms through which *Francisella novicida* inhibit apoptosis (34). However, it is not clear whether CPSIT_0556 can produce IL-8 or whether IL-8 regulates these apoptosis-related proteins.

In this study, we aimed to explore the effect of CPSIT_0556 on hPMN apoptosis and further clarify the potential mechanism, hoping to provide a new insight for the escape of immune clearance of *C. psittaci* by hPMN.

## Materials and Methods

### Antibodies and Reagents

Rabbit anti-phosphorylated and anti-total p38MAPK mAb, rabbit anti-phosphorylated and anti-total IκB mAb, rabbit anti-phosphorylated and anti-total AKT mAb, rabbit anti-Mcl-1 mAb, rabbit anti-procaspase 3 mAb, mouse anti-active caspase-3 mAb, mouse anti-β-actin mAb, rabbit anti-IgG, and mouse anti-IgG were purchased from Cell Signaling Technology (Beverly, MA, USA). Mouse anti-His mAb was purchased from Affinity Biosciences, Inc (OH, USA). The rabbit anti-*C. psittaci* mAb was a gift from Professor G. Zhong (University of Texas Health Science Center at San Antonio, TX). The rabbit anti-SOD2 antibody, mouse anti-Bax antibody was purchased from ImmunoWay Biotechnology Company (Plano, TX, USA). The inhibitors SB203580, BAY117082, LY294002, staurosporine (STS), and Cy2-conjugated goat anti-rabbit IgG, DAPI, Cy3-conjugated goat anti-mouse IgG, and Cy3-conjugated goat anti-rabbit IgG and mouse anti-NF-κB p65 antibody were obtained from Abcam (Cambridge, MA, USA), recombinant human IL-8 from (Biolegend, CA, USA), anti-IL-8 mAb was obtained from GeneTex, USA.

### Ethics Statement

All animal studies were approved by the Animal Welfare Committee of the University of South China and conducted in accordance with university regulations to minimize animal suffering. Mouse care and monitoring followed the protocol approved by the University of South China Institutional Animal Use and Ethics Committee (Hengyang, China).

All human subjects provided informed consent before participating in the study and provided written informed consent. This study was approved by the Ethics Committee of the University of South China and is in accordance with the Declaration of Helsinki.

### *C. psittaci* Propagation and Purification

HeLa cells (ATCC, CCL-2.1) were cultured for 16–18 h at 37°C and 5% CO_2_. When the cell density reached about 60%–80%, The *C. psittaci* 6BC (ATCC VR-125) infection solution was diluted with DMEM (HyClone, USA). After washing HeLa cells with PBS, the prepared infectious fluid was inoculated into a cell culture flask, and incubated at 37°C with 5% CO_2_, and gently shaken once every 20 min to evenly distribute the infectious fluid in each cell. A moderate amount of actinone was then added to complete medium containing 10% FBS (Excell Bio, Uruguay). After 48 h, the 400 μL of trypsin (Solarbio, China) was taken to digest the cells. Digestion was stopped by exposure to complete medium, HeLa cells were collected in a centrifuge tube at 4°C, 1000× rpm for 10 min. The supernatant was discarded, then add 200 μL of SPG solution (dodecahydrate disodium hydrogen phosphate 308 mg, potassium dihydrogen phosphate 52 mg, L-glutamic acid 72 mg, and sucrose 7500 mg, dissolved in 100 mL deionized water at pH 7.4–7.6, filtered and sterilized), EBs were then purified by density gradient centrifugation (35), and stored at −80°C until use.

### Expression, Purification, and Serum Preparation of Recombinant CPSIT_0556

Since inclusion membrane proteins are difficult to purify, and our experiments have confirmed that N-terminal has no effect on CPSIT_0556 inclusion membrane localization and hPMN apoptosis (data not shown). Therefore, we intercepted C-terminal, the forward primer for CPSIT_0556 was 5’-ATGTCTCATTTCTATTTGATGCAGCATG-3’, and the reverse primer was 5’-TTATCCGACAAAATCAAGTTCTTCTGAG-3’, then was cloned into the expression vector pET-28a, and the construct was transformed into *E. coli* BL21. Using IPTG (Biosharp, China) was induce used to induce expression in *E. coli* BL21. The non-target protein was eluted with different concentrations of imidazole wash buffer. Treatment with polymyxin B (Sigma, USA) removed endotoxin contamination, and endotoxin levels were measured with Limulus amebocyte lysate (Xiamen Limulus Reagent Co., Ltd., China). Endotoxin levels less than 0.03 endotoxin units (EU)/mL were considered acceptable.

Pathogen-free BALB/c mice (6-8 weeks of age) were purchased from Hunan Saike Jingda Experimental Animal Co., Ltd. BALB/c mice were injected once with 100 μg recombinant CPSIT_0556 protein emulsifying compound and complete Freund’s adjuvant. Mice were immunized with the same dose of protein and incomplete Freund’s adjuvant (Sigma, USA) on days 14 and 28, if necessary, they were immunized twice. Blood was collected from the tail before each immunization, and the antibody titer was detected by ELISA. After the last immunization, a blood sample was collected to prepare polyclonal serum, which was stored at -80°C for use.

### Preparation and Co-Incubation of hPMN With CPSIT_0556

Blood samples were donated by healthy volunteers with no underlying disease who had not taken medication the week before the collection. Blood was collected with a purple anticoagulant tube containing EDTA and gently mixed by inversion to prevent blood clotting. A 5 mL volume of Polymorphprep™ (Axis-Shield, Norway) and the same volume of blood was transferred to a 15 mL sterile enzyme-free centrifuge tube, and the tubes were centrifuged at 500 ×g room temperature for 30 min. The resulting first layer was rich in lymphocytes and monocytes, which was carefully removed and discarded. The second layer was rich in hPMN, which was collected and transferred to a 15 mL sterile enzyme-free centrifuge tube, washed with the same amount of PBS, and then centrifuged at 400 ×g for 10 min. Red blood cell (RBCs) lysis buffer (Biosharp, China) was added to the pellet to lyse any residual RBCs for 3–5 minutes, followed by washes with PBS for 10 min at 400 ×g. The hPMN obtained were resuspended in RPMI 1640 medium supplemented with 10% FBS and 10 mM HEPES. The purity of hPMN cells obtained by this separation technique remained stable for each extraction, and the separation purity was evaluated by Giemsa staining. hPMN activity was assessed by trypan blue staining, the separation purity of hPMN achieved using this method is greater than 98%.

HPMN (2 × 10^6^ cells/mL) were cultured in 1640 containing 10% FBS and then incubated with CPSIT_0556 for 12, 24, and 36 h. Staurosporine-treated was used as a positive control. The cell cultures were maintained in 5% CO_2_ and 37°C incubators. The PI3K/Akt, NF-κB, and p38 MAPK pathway inhibitors used were LY294002, BAY 11-7082, and SB203580, respectively, which were incubated with cells for 30 minutes in advance as pre-treatment conditions, before the addition of CPSIT_0556 for the specified incubation time. Further co-culture experiments were conducted using recombinant human IL-8, with supernatants taken from hPMN-CPSIT_0556 cultures or with the same supernatants after IL-8 depletion.

### Annexin-V Binding

Apoptosis was determined by using the Annexin V-FITC/PI Kit (UE, China) according to the manufacturer’s recommendations. Briefly, hPMN were centrifuged at 300 ×g for 5 min, washed with PBS, and centrifuged at 350 ×g for 5 min. A 100 μL volume of 1× binding buffer, 3 μL FITC-Annexin, and 5 μL PI were added to the sample and incubated in the dark for 15 min. Each sample was shaken once every 5 min, and then 400 μL 1× binding buffer was added to the sample tube. hPMN were identified by BD FACS Canto II flow cytometer (Becton Dickinson, USA) and the experimental data were collected for FlowJo 7.6.1 software analysis (BD Biosciences, USA). For inhibitor experiments, before the addition of CPSIT_0556, hPMN were pre-incubated for 30 min with PI3K inhibitor LY294002, NF-κB inhibitor BAY 11-7082, and the p38 MAPK inhibitor SB203580.

### Immunoblotting Analysis

HPMN were grown in 12-well plates at a density of 1-5×10^6^ cells/well. Each sample was centrifuged at 1000 rpm at 4°C, for 10 minutes. Centrifuged cells were washed once with cold PBS and RIPA lysis buffer containing phosphatase inhibitors and protease inhibitors was added. After incubation in ice for 1 h, the whole-cell lysates were centrifuged at 4°C at 13,000 ×g for 10 min. Proteins were measured using the BCA Protein Assay Kit (Thermo Fisher, USA). Equal amounts of sample protein were added with the same proportion of SDS sample loading buffer and boiled at 100°C for 5–10 minutes before loading onto SDS-PAGE gels of 12.5% or 15%, prepared based on the molecular weights of the target proteins. After electrophoresis, proteins were transferred onto PVDF membrane (Millipore, Bedford, MA, USA) using the appropriate aperture. Methanol permeation of the PVDF membrane was required for 10 s, 2 min before the membrane transfer. After membrane transfer, the PVDF membrane was immersed in TBST containing 5% non-fat milk for 2 h at room temperature. The PVDF membrane was incubated with diluted primary antibody at 4°C overnight, washed several times with TBST, and then incubated with the secondary antibody for 1 h.

### Immunofluorescence

HPMN were grown in 12-well plates at a density of 2 × 10^6^ cells/well. After washing with PBS, cells were fixed in 4% paraformaldehyde (Biosharp, China) at room temperature for 1 h, and permeated with 0.3% Triton X-100 at 4°C for 20 min. Next, cells were blocked with RPMI 1640 medium containing 10% FBS for 1 h, and then washed and incubated overnight with mouse anti-NF-κB p65 antibody (1:200 dilution) or rabbit anti-SOD2 antibody (1:50 dilution) or mouse anti-Bax antibody (1:50 dilution). Finally, cells were incubated with Cy2-conjugated goat anti-rabbit (1:200 dilution) or Cy3-conjugated goat-anti-mouse antibody (1:200 dilution) for 1 h. After washing with PBS and staining with 4’,6-Diamidino-2-phenylindole dihydrochloride (DAPI, 1 mg/mL) for 10 min for staining nuclei.

### IL-8 ELISA Assay

The culture supernatants of hPMN were determined by ELISA assays using a commercially available kit for IL-8 (Thermo Fisher, USA), following the manufacturer’s instructions.

### Statistical Analysis

The results of all experiments were reported as the mean ± SEM of at least three independent experiments. Statistical analyses were performed using GraphPad Prism 5.0 software (GraphPad Software, Inc., La Jolla, CA). Differences were considered significant at a *P-*value < 0.05.

## Results

### Expression, Purification, and Localization of CPSIT_0556

**Figure 1:**
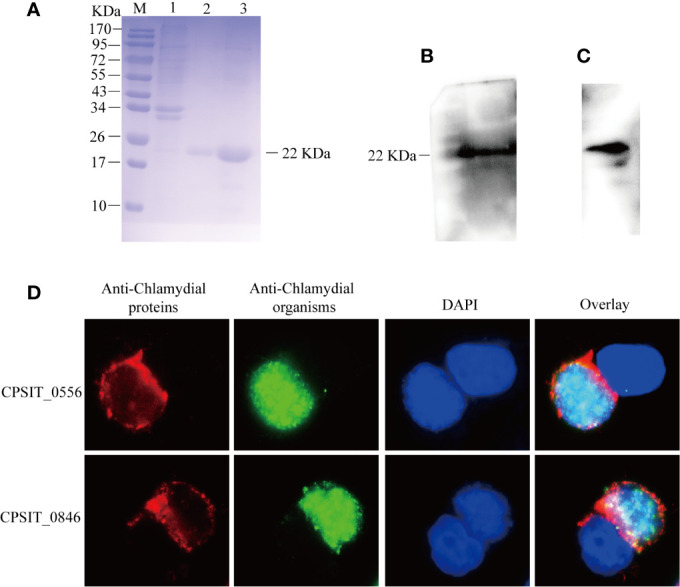
Purification of CPSIT_0556 and subcellular localization in *C. psittaci*-infected HeLa cells. **(A)** CPSIT_0556 protein was separated by 12.5% SDS-PAGE, Lane 1: CPSIT_0556 in *E. coli* BL21 without IPTG, Lanes 2-3: Lanes 2–3: 300, 500 mM imidazole wash buffer, respectively. The recombinant CPSIT_0556 protein with a predicted size of 22 kDa. **(B)** CPSIT_0556 expression analyzed by Western blotting using the rabbit anti-*C. psittaci* mAb. **(C)** CPSIT_0556 analyzed by Western blotting using the anti-His labeled mAb. **(D)** *C*. *psittaci* 6BC infected HeLa cells for 48 h and then cells were probed with rabbit anti-*C. psittaci* mAb, mouse anti-CPSIT_0556, or mouse anti-CPSIT_0846 (red), the second antibody was Cy2-conjugated goat anti-rabbit IgG, Cy3-conjugated goat anti-mouse IgG, and DAPI. CPSIT_0846 was the positive control for inclusion membrane protein (×1000, in oil).

It has been reported that Incs all share a common characteristic structure consisting of a hydrophobic bilobate domain (36). CPSIT_0556 is characterized by a bifoliate hydrophobic motif, which is consistent with the characteristics of inclusion membrane proteins CPSIT_0846. The purity of CPSIT_0556 protein was successfully expressed in *E. coli* BL21 as a soluble fusion protein with a predicted size of 22 kDa. The purity of eluted proteins was analyzed by SDS-PAGE, then determined by BandScan 5.0 software, and analysis revealed it to have a purity of >95% (**Figure 1A**). The CPSIT_0556 fusion protein was identified by immunoblotting with an anti-*C. psittaci* and anti-His tag monoclonal antibody (**Figures 1B, C** **)**. The localization of CPSIT_0556 in HeLa cells infected with *C. psittaci* was detected by immunofluorescence assay. CPSIT_0556 was localized in the inclusion membrane (**Figure 1D**), which is similar to the *C. psittaci* inclusion membrane protein CPSIT_0846 (6).

### CPSIT_0556 Delayed Spontaneous hPMN Apoptosis

**Figure 2:**
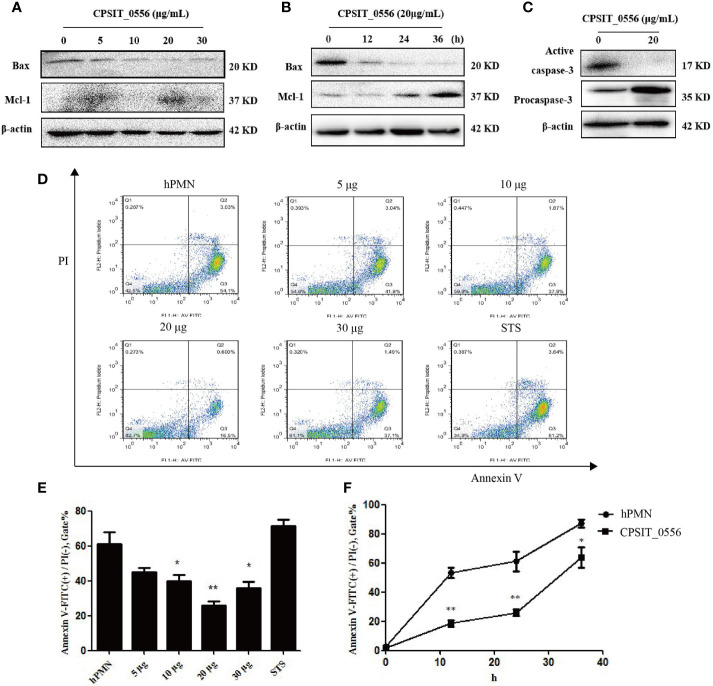
CPSIT_0556 delays spontaneous hPMN apoptosis. **(A)** CPSIT_0556 stimulated hPMN at different concentrations for 24 h, expressions of Bax and Mcl-1 were detected by Western blotting. **(B)** Exposure of hPMN to CPSIT_0556 at different times; expression of Bax and Mcl-1 were detected by Western blotting.**(C)** Expression of procaspase-3 and active caspase-3 in hPMN with or without CPSIT_0556 for 24 h. **(D)** Exposure of hPMN to CPSIT_0556 at different concentrations for 24 h, and the apoptosis rate was detected by flow cytometry. **(E, F)** Apoptosis rate of hPMN with or without CPSIT_0556 at different concentrations and at different times. *P < 0.05, **P < 0.01.

*In vitro*, hPMN cells have a short life span and spontaneous apoptosis occurs within 6–10 h (37). Studies have shown that *Chlamydia* spp. can resist apoptosis of host cells in the early stages of growth and development, while in the late stages of *Chlamydia* spp. infection promote their proliferation by inducing host cell apoptosis (38). Thus, Chlamydia resistance to host cell apoptosis is an important mechanism to achieve immune escape (16). Previous studies have shown that hPMN do not express Bcl-x or Bcl-2, but constitutively express Bax and Mcl-1 (39, 40). To investigate whether the inclusion membrane protein CPSIT_0556 of *C. psittaci* can inhibit the apoptosis of hPMN, we evaluated the expression of Bax and Mcl-1 following CPSIT_0556 culture with stimulated hPMN at different concentrations and at different timepoints. Western blotting experiments showed that CPSIT_0556 inhibited hPMN apoptosis most notably at 20 μg/mL, but the inhibition was weakened at higher concentrations 30 μg/mL (**Figure 2A**). Thus, we stimulated hPMN at 20 μg/mL of CPSIT_0556 at for different timepoints, and the results showed that CPSIT_0556 could up-regulating the expression of anti-apoptotic protein Mcl-1 and down-regulating the expression of pro-apoptotic protein Bax in hPMN (**Figure 2B**).

To further understand the mechanisms responsible for CPSIT_0556 prolonging hPMN lifespan, we explored the effects of CPSIT_0556 on the core components of the apoptosis pathway. Caspase-3 is an inactive 35 kDa precursor enzyme that is processed into a mature/active (17–19 kDa) form following upstream caspase activation and signal transduction, which is a major effector in hPMN, responsible for the cleavage of cell substrates, and leads to biochemical and morphological changes specific to apoptotic cells. These changes include PS externalization, nuclear condensation, and DNA fragmentation (41). Moreover, caspase-3 is considered an important regulator involved in apoptosis, and its activation is inhibited when cells are infected with Chlamydia (42). However, it is not clear whether CPSIT_0556 stimulation of hPMN inhibits caspase activation. Freshly isolated hPMN cells contain high levels of procaspase-3, which is cleaved during spontaneous apoptosis to produce enzyme-active caspase-3 (43). In our study, procaspase-3 was activated in isolated hPMN after a 24-h culture. Instead, CPSIT_0556 inhibited the activation of caspase-3 (**Figure 2C**).

Next, by using flow cytometry, we stimulated hPMN with different concentrations of CPSIT_0556 for 24 h, which was consistent with the Western blot results, and the most obvious was 20 μg/mL stimulation (**Figure 2D**). The histogram shows flow cytometry statistics of CPSIT_0556 stimulated hPMN at different concentrations and at different times (**Figures 2E, F**).

### PI3K/Akt and NF-κB Activities Were Required for the CPSIT_0556 Inhibition of hPMN Apoptosis

**Figure 3:**
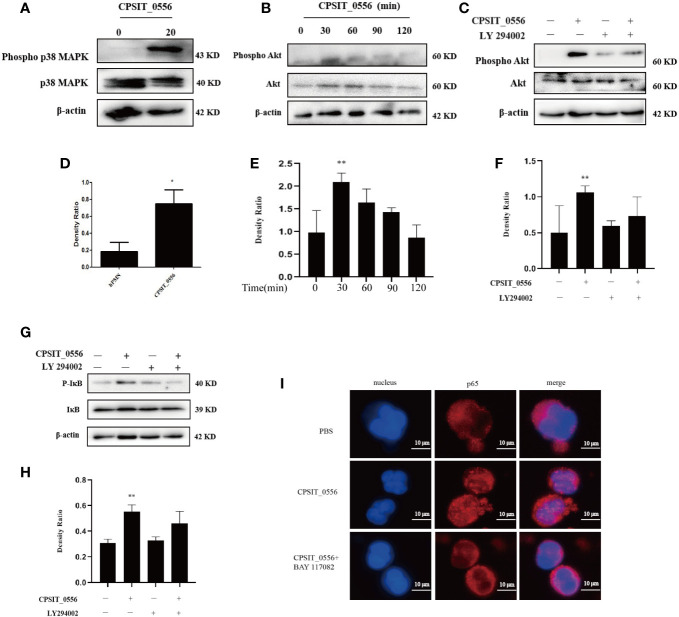
CPSIT_0556 activates the p38 MAPK, PI3K/Akt, and NF-κB pathways in hPMN. **(A, D)** HPMN was cultured with 20 μg/mL CPSIT_0556 for 1 h, the phosphorylation of p38 MAPK was analyzed by Western blotting and quantified using ImageJ software. **(B, E)** HPMN was cultured with CPSIT_0556 with for different time points, the phosphorylation of Akt was analyzed by Western blotting and quantified using ImageJ software. **(C, F)** HPMN were pretreated with 25μM LY294002 for 30 min, and then incubated with CPSIT_0556 for 30 min, the phosphorylation of Akt was analyzed by Western blotting and quantified using ImageJ software. **(G, H)** HPMN were incubated with or without the PI3K inhibitor LY294002 (25 μM) for 30 min and then cultured with or without 20 μg/mL CPSIT_0556 for 30 min. the phosphorylation of IκB was analyzed by Western blotting and quantified using ImageJ software, *P < 0.05 *vs*. control groups, **P < 0.01 *vs*. control groups. **(I)** HPMN were stimulated with CPSIT_0556 (20 μg/mL) for 12 h and stained with mouse anti-p65 mAb (1:200 dilution), and then incubated with Cy3-conjugated goat anti-mouse IgG (1:200 dilution). DAPI staining of the nucleus, the image was obtained with an optical microscope (×1000, in oil).

Previous studies have shown that p38 MAPK constitutes a survival signal in hPMN (44). Therefore, we investigated the phosphorylation of p38 MAPK in CPSIT_0556-stimulated hPMN. Western blotting analysis showed that p38 MAPK was phosphorylated in CPSIT_0556 stimulated hPMN at 1 h, while the phosphorylation of p38 MAPK in hPMN was not longer present (**Figures 3A, D** **)**. Moreover, the PI3k signaling pathway plays an important role in hPMN (45). Previous studies have shown that the PI3K/AKT pathway was associated with the resistance to apoptosis of hPMN infected with *Chlamydia pneumoniae* and *Anaplasma phagocytophilum* (46, 47); however, whether CPSIT_0556 activates this pathway is unknown. Western blot analysis revealed that stimulation with CPSIT_0556 resulted in the phosphorylation of Akt in hPMN, which most markedly at 30 min (**Figures 3B, E** **)**.

The enhancement of Akt phosphorylation in CPSIT_0556-stimulated hPMN suggested that the activation of this pathway may be involved in the delay of apoptosis induced by CPSIT_0556. Since other methods such as siRNA are not feasible for hPMN, we used pharmacological inhibitors to determine whether the PI3K/Akt pathway plays a role in CPSIT_0556-induced apoptosis delay. We pretreated hPMN with the selective PI3K inhibitor LY294002 to block the phosphorylation of PI3K kinase-dependent protein kinase. Western blotting analysis showed that this treatment inhibited the phosphorylation of Akt induced by CPSIT_0556 (**Figures 3C, F**).

To further verify the activation of NF-κB in CPSIT_0556-stimulated hPMN, Western blotting was used to evaluate the phosphorylation status of IκB. The results showed that CPSIT_0556 stimulated hPMN phosphorylation of IκB was higher than that of hPMN cells cultured in the absence of CPSIT_0556 (**Figures 3G, H**). Activated Akt promotes cell survival by phosphorylation of several downstream proteins, including those associated with the cell survival/death pathway, such as Bax, Mcl-1, and NF-κB (46, 48). In hPMN cells, however, it is not clear whether these pathways are related. Next, we investigated the relationship between PI3K/Akt and NF-κB signaling in CPSIT_0556-stimulated hPMN. Western blotting analysis showed that CPSIT_0556-induced NF-κB activation was significantly inhibited when exposed to the PI3K inhibitor LY294002 (**Figures 3G, H**), suggesting that the PI3K/Akt pathway was involved in CPSIT_0556-mediated NF-κB activation.

NF-κB is a nuclear transcription factor with multiple regulatory activities that involve various pathophysiological processes such as inflammation, immunity, cell proliferation, and apoptosis (49). During activation of NF-κB, polyubiquitination of the inhibitory phosphorylated-IκB subunit is inhibited, leading to its dissociation, which allows free cytoplasmic transport of NF-κB to the nucleus (50). The nuclear localization of NF-κB in hPMN exerts a strong anti-apoptotic signal (51). Therefore, we used the immunofluorescence to explore whether the NF-κB signaling pathway was activated after CPSIT_0556 stimulation of hPMN. The results showed that in hPMN cultures without CPSIT_0556, the NF-κB immunofluorescence signal was distributed in the cytoplasm; however, exposure to CPSIT_0556 significantly promoted the nuclear translocation of NF-κB. Furthermore, nuclear translocation of NF-κB disappeared in hPMN co-cultures pre-treated with BAY11-7082 **(** **Figure 3I**).

**Figure 4:**
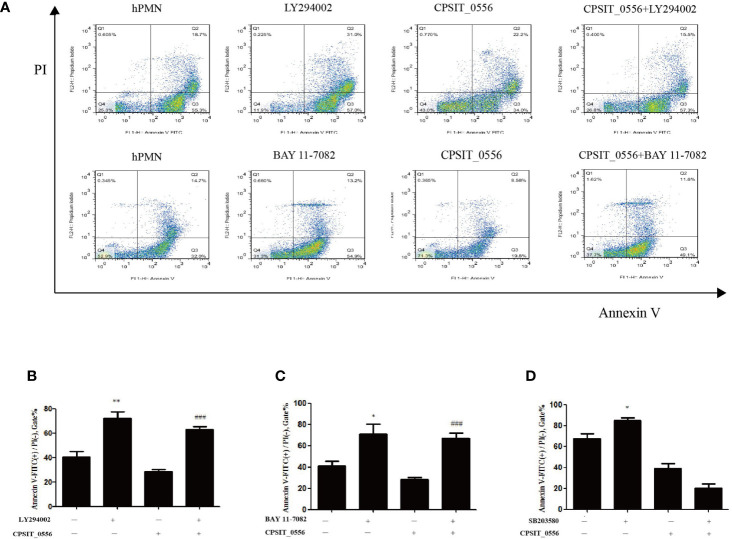
CPSIT_0556 inhibits hPMN apoptosis through PI3K/AKT and NF-κB signaling Pathways. **(A)** HPMN pretreated with 25 μM LY294002 or 10 μM BAY 11-7082 for 30 min, and then incubated with CPSIT_0556 for 24 h. **(B, C)** The bar chart shows flow cytometric statistics for LY294002 or BAY11-7082 treatment of hPMN. The apoptosis rate of at least 20,000 cells was determined by flow cytometry of at least 20,000 cells in three separate trials. *P < 0.05 compared to hPMN cultured separately. **P < 0.01 compared to hPMN cultured separately. ^###^ *P* < 0.001 between CPSIT_0556-stimulated hPMN with and without the inhibitor BAY 11-7082 or LY294002. **(D)** HPMN were pretreated with p38 MAPK inhibitor SB203580 (10 μM) for 30 min, then incubated with or without CPSIT_0556 for 24 h; data show the percentage of apoptotic cells determined by flow cytometry of at least 20,000 cells in three separate trials, *P < 0.05 compared to hPMN cultured separately.

CPSIT_0556 activates the p38 MAPK and PI3K/Akt pathways of hPMN, but whether these pathways are involved in the inhibition of hPMN apoptosis by CPSIT_0556 remains unclear. Flow cytometry showed that compared with the control group, the apoptosis rate of hPMN stimulated by PI3K inhibitor LY2904002 was increased. In addition, treatment with NF-κB inhibitor BAY11-7082 increased the apoptosis rate of hPMN, and both LY294002 and BAY11-7082 inhibited the delay of apoptosis induced by CPSIT_0556 (**Figure 4A**). The histogram shows flow cytometric statistics of these two inhibitors at hPMN (**Figures 4B, C**). SB203580 is an inhibitor of the p38 MAPK pathway. Treatment with SB203580 did not affect the delay in CPSIT_0556-induced apoptosis (**Figure 4D**). These findings suggested that although CPSIT_0556 enhanced p38 MAPK phosphorylation, this event was not directly involved in the induced delay in apoptosis of hPMN.

### CPSIT_0556 Promoted hPMN Survival by Regulating the Expression of Bax and Mcl-1 Through PI3K/Akt Signaling

**Figure 5:**
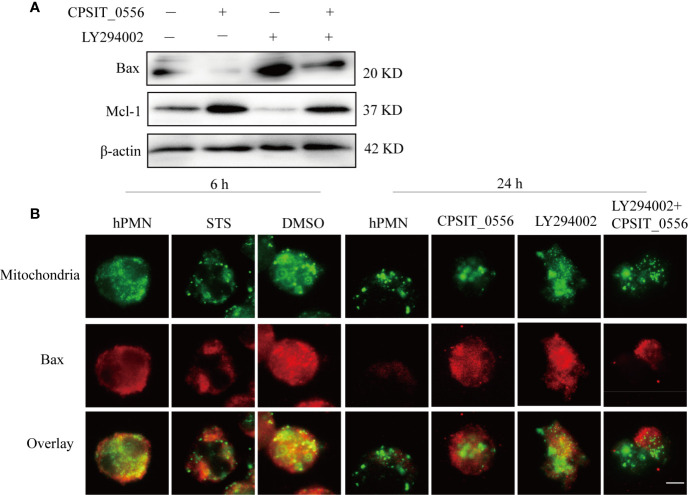
Activation of the PI3K/Akt pathway inhibits Bax from entering the mitochondrial membrane. **(A)** HPMN were pre-treated with the PI3K inhibitor LY294002 (25μM) for 30 min and then cultured with 20 μg/mL CPSIT_0556 for 6 h. Western blotting showing the expression levels of Bax and Mcl-1. **(B)** HPMN were left untreated, treated with DMSO or STS as indicated for 6 h, or treated with CPSIT_0556, 25 μM LY294002, LY294002 was co-incubated with CPSIT_0556 for 24 h, stained with rabbit anti-SOD2 monoclonal (1:50 dilution) and mouse anti-Bax monoclonal (1:50 dilution), then incubated with Cy3-conjugated goat anti- mouse IgG (1:200 dilution) and Cy2-conjugated goat anti-rabbit IgG (1:200 dilution). DAPI staining of the nucleus, the cytoplasmic distribution of mitochondria (green fluorescence), and Bax (red fluorescence) were detected by double immunofluorescence labeling. Imaging results were observed by an optical microscope (×1000, in oil). The scale bar is 5 µm, the image represents at least three separate experiments.

HPMN viability is influenced by a variety of signals, including pro-apoptotic signals and pro-survival signals (52). Bax is considered a quantitative marker of early apoptotic events (29, 31, 53). Anti-apoptotic stimulation inhibits the insertion of Bax into the mitochondrial membrane, thereby inhibiting its pro-apoptotic activity (31). Conversely, during hPMN cell death, the level of Mcl-1 protein decreases gradually, causing Bax to be released from Mcl-1 (26). The balance between anti-apoptotic and pro-apoptotic molecules determines the fate of the cell (54). Previous studies have shown that the Bax/Mcl-1 balance can affect hPMN survival during intermittent hypoxia and obstructive sleep apnea (55). Thus, we explored the expression of Bax/Mcl-1 in hPMN stimulated by CPSIT_0556 and the influence of the PI3K/Akt pathway on its expression. Following CPSIT_0556 stimulation of hPMN, the expression of Bax was down-regulated and the expression of Mcl-1 was up-regulated. In contrast, following pretreatment with the PI3K inhibitor LY294002, the expression of Bax was up-regulated and the expression of Mcl-1 was down-regulated (**Figure 5A**).

To further investigate whether CPSIT_0556 and PI3K/AKT signaling are involved in inhibiting the insertion of Bax into the mitochondrial membrane and thereby inhibiting its pro-apoptotic activity, the expression of Bax and SOD2 was detected by immunofluorescence assay. SOD2 is mainly located in the mitochondria of prokaryotic cells and eukaryotic cells. When hPMN were cultured *in vitro* for 6 h, Bax presented a diffuse cytoplasmic localization and was not inserted into the mitochondrial membrane. STS was used as a positive control and DMSO as the solvent control. At 24 h, exposure to CPSIT_0556 inhibited the insertion of Bax into mitochondrial membrane, while after treatment with PI3K inhibitor LY294002, Bax was distributed into mitochondria. Conversely, following pretreatment with the PI3K inhibitor LY294002 for 30 min, CPSIT_0556 was added to the culture, and Bax was gradually redistributed from mitochondria to the cytoplasm (**Figure 5B**). These results indicated that CPSIT_0556 inhibited the insertion of Bax into the mitochondrial membrane in hPMN, thereby inhibiting its pro-apoptotic activity, and the PI3K/AKT signaling played an important role in this process.

### CPSIT_0556 Induced IL-8 Production of hPMN

**Figure 6:**
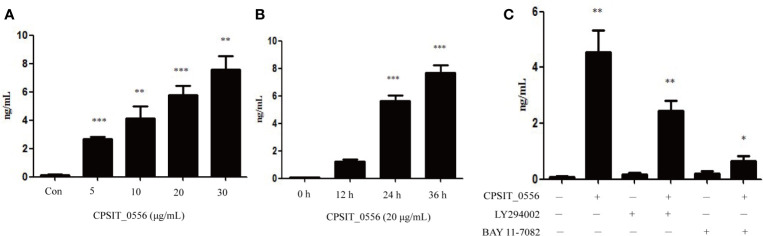
CPSIT_0556 induced the release of IL-8 from hPMN. **(A)** hPMN were exposed to CPSIT_0556 at different concentrations or in medium alone for 24 h. **(B)** hPMN were co-cultured with 20 μg/mL CPSIT_0556 for 12, 24, and 36 h. **(C)** hPMN were pre-incubated with or without inhibitors of NF-κB (10 μM BAY 11-7082) or with the PI3K inhibitor LY294002 (25 μM) for 30 min before co-incubation with CPSIT_0556 for 24 h. IL-8 levels were measured using ELISA. *P < 0.05, **P < 0.01, ***P < 0.001.

IL-8 are generally considered take part in a positive feedback amplifying response used to recruit hPMN to acute infection/inflammation sites (56). Previous studies have shown that *C. pneumoniae* (15), *Leptospira* (57), and *Anaplasma phagocytophilum* (46) can induce hPMN to produce IL-8. Therefore, we investigated whether CPSIT_0556 can induce hPMN to produce inflammatory cytokines, such as IL-8. The results showed that different concentrations of CPSIT_0556 could induce IL-8 production by hPMN. When stimulated by 30 μg/mL, the production of IL-8 by hPMN was the highest, while the levels of IL-8 produced by hPMN cultured without CPSIT_0556 were very low (**Figure 6A**). At the same time, when hPMN were exposed to a concentration of 20 μg/mL CPSIT_0556 for 12, 24, and 36 h, the secretion of IL-8 was gradually increased (**Figure 6B**). The above results showed that CPSIT_0556 induced IL-8 production of hPMN in a dose- and time-dependent manner.

Next, we explored which pathways are activated by CPSIT_0556 in hPMN to release IL-8. Activation of NF-κB has been shown to lead to the release of IL-8 in hPMN (58); thus, we explore whether CPSIT_0556-induced IL-8 release was regulated by the NF-κB signaling pathway. Our results showed that treatment with the IκB inhibitor significantly reduced CPSIT_0556-induced IL-8 release. Furthermore, in the hPMN cultured without CPSIT_0556 but in the presence of the IκB inhibitor BAY 11-7082, IL-8 was almost not secreted. These results suggested that CPSIT_0556-induced IL-8 release by hPMN dependent on the NF-κB signaling pathway (**Figure 6C**). PI3K phosphorylation is an upstream signaling event in the NF-κB pathway of CPSIT_0556-stimulated hPMN. Therefore, we investigated whether CPSIT_0556-induced hPMN could regulate IL-8 production through the PI3K/Akt pathway. Treatment with the PI3K inhibitor LY294002 significantly reduced IL-8 release in CPSIT_0556-stimulated hPMN (**Figure 6C**).

### IL-8 Activated PI3K/Akt Signaling and Up-Regulated the Expression of Mcl-1 in hPMN

**Figure 7:**
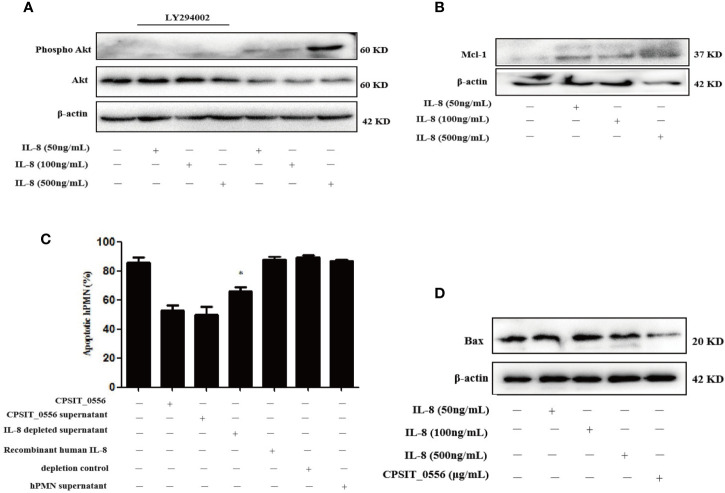
IL-8 enhances Akt phosphorylation through the PI3K-dependent pathway as well as increases the expression of Mcl-1. **(A)** HPMN were pre-treated with the PI3K inhibitor LY294002 (25 μM) for 30 min., then treated with recombinant human IL-8 (50 ng/mL and 100 ng/mL and 500 ng/mL) for 30 min. Western blotting showing the phosphorylation of AKT. **(B)** HPMN were incubated with recombinant human IL-8 for 30 min. Western blotting showing the expression of Mcl-1, β-actin served as the loading control. The data shown are from one experiment and represent three independent experiments. **(C)** HPMN were cultured in medium alone, or co-cultured with CPSIT_0556, with supernatant of CPSIT_0556-hPMN, with these same supernatants depleted for IL-8 using a polyclonal anti-IL-8 mAb and protein A-agarose immunoprecipitation reagent, with recombinant human IL-8 (50 ng/mL), with a depletion control, or with supernatant from hPMN after 24 h. The apoptosis rate of hPMN was analyzed by flow cytometry after a 24-h culture, the data represent at least three separate experiments.*P < 0.05 compared to CPSIT_0556 supernatants prior to IL-8 depletion. **(D)** The hPMN were cultured in different concentrations of IL-8 for 24 h, and the expression of Bax was detected by Western blot, the data shown are from one experiment and represent three independent experiments.

Our results showed that CPSIT_0556 not only induced the secretion of IL-8 by hPMN, but also inhibited the apoptosis of hPMN through the PI3K/Akt signaling pathway. Most studies have shown that IL-8 can inhibit the apoptosis of hPMN (40, 59), but the mechanism through which IL-8 inhibits apoptosis remains unclear. Therefore, we explored whether IL-8 could inhibit the apoptosis of hPMN through the PI3K**/**Akt pathway. Cytokines have a short biological half-life, thus we exposed hPMN to different concentrations of IL-8 for 30 min. Western blotting results showed that hPMN exposure to IL-8 induced Akt phosphorylation, and in turn IL-8-induced Akt phosphorylation was completely reduced when hPMN were pre-treated with the PI3K inhibitor LY294002 (**Figure 7A**).

Since IL-8 activates the PI3K/Akt pathway, while Mcl-1 is dependent on the PI3K/Akt pathway for its activity, we tested the effects of IL-8 on Mcl-1 expression. Mcl-1 expression increased in IL-8-exposed hPMN at 30 min (**Figure 7B**). *C. pneumoniae* LPS has been reported to promote the release of IL-8 from hPMN, thereby inhibiting the apoptosis of hPMN (15). In the study investigating *F. novicida*, recombinant human IL-8 was found to induce transient inhibition of apoptosis, which was statistically significant at 12 h, and gradually decreased at 24 h (34). Thus, we tested whether IL-8 produced by hPMN after CPSIT_0556 stimulation could inhibit hPMN apoptosis. IL-8 was depleted from the supernatant using an anti-IL-8 mAb and protein A-agarose immunoprecipitation reagent (depletion control). HPMN were cultured in medium alone, hPMN exposed to CPSIT_0556, hPMN cultured with supernatant of CPSIT_0556-hPMN, hPMN cultured in the supernatant of CPSIT_0556-hPMN depleted for IL-8 as indicated above, or cultured with recombinant human IL-8, with a depletion control, or with supernatant obtained from hPMN after a 24 h culture. The apoptosis rate of each group was analyzed by flow cytometry. The apoptosis rate of the hPMNs treated with CPSIT_0556 cultured with supernatant decreased, the apoptosis rate hPMNs cultured in IL-8-depleted medium was higher than that of the hPMNs treated with CPSIT_0556 with un-depleted IL-8 supernatant, while the recombinant human IL-8 had no effect on the apoptosis of hPMN (**Figure 7C**). Western blotting results also showed that recombinant human IL-8 at different concentrations could not down-regulate the expression of Bax (**Figure 7D**). Altogether, IL-8 anti-apoptotic effect may require cooperation with other cytokines, which needs further study.

## Discussion

Our study demonstrated for the first time that CPSIT_0556 plays an important role in inhibiting the apoptosis of hPMN. Further, the study revealed the main pathways involving of CPSIT_0556-mediated hPMN apoptosis delay, which may be related to intrinsic apoptosis pathways. Studies have shown that the PI3K/Akt signaling pathway plays a major role in CPSIT_0556 inhibition of apoptosis of hPMN. As an upstream pathway, the PI3K/Akt signaling pathway is not only involved in regulating the expression of Bax and Mcl-1, but also regulates the activation of the NF-κB pathway in hPMN. Furthermore, CPSIT_0556 was able to inhibit the entry of Bax into the mitochondrial membrane and to induce p65 transfer from the cytoplasm to the nucleus to inhibit apoptosis.

The complex role of hPMN in severe pneumonia highlights specific molecules and processes that promote lung immunity but can also contribute to the progression of severe disease (9). During tularemia, hPMN contributes to disease progression rather than to effective disease defense, thus, manipulating hPMN migration or transformation may be an appropriate adjuvant therapeutic strategy (60). Inclusions provide a protective intracellular microenvironment for Chlamydia replication, which allows it to evade the host’s surveillance and innate immune responses. Extensive modification of the inclusion membrane by the insertion of multiple inclusion membrane proteins at the early stage of infection underscores the importance of this closed compartment (61). During the intracellular growth phase of Chlamydia, maintaining the integrity of host cells is not only necessary to provide nutrients, but also protects intracellular microorganisms from various adverse growth factors (16). When *Chlamydia pneumoniae* invade the hPMN, the inclusion formed by EBs and RBs in hPMN differed from those formed on invasion of epithelial cells, but were similar to those observed monocytes (62), which suggested that inclusion formation is an important mechanism used by Chlamydia to escape hPMN killing. Due to limitations of experimental techniques, the CPSIT_0556 gene locus could not be targeted for knock-out studies or to construct *C. psittaci* specific locus mutants. Therefore, we relied on the prokaryotic expression of CPSIT_0556 to explore the mechanisms involved in inhibiting hPMN apoptosis. Since it is difficult to purify 100% recombinant protein (63), to make the experimental design more rigorous, we purified another inclusion membrane protein CPSIT_0842 by the same method before and found that CPSIT_0842 could not inhibit hPMN apoptosis (data not shown), which indicated that CPSIT_0556 had a specific inhibitory effect on hPMN apoptosis, and the low contamination of bacterial protein seems not to influence the CPSIT_0556-induced apoptosis delay. However, the purified protein is prone to degradation in a non-low temperature environment, and we cannot completely rule out whether the impurity protein affects hPMN apoptosis, so a more optimized protein purification scheme remains to be explored.

Compared with mechanisms of observed in other *Chlamydia* spp. the pathogenesis of *C. psittaci* is relatively unknown. It is often misdiagnosed due to its insidiousness and atypical clinical symptoms, which are similar to other respiratory pathogens (64). Thus, it is important to be aware of the dangers of Chlamydia infection and ensure a timely diagnosis. Chlamydia regulates apoptosis by taking advantage of host cell mechanisms (16). PI3K/Akt, p38 MAPK, and NF-κB pathways are the main survival signaling pathways in hPMN (24, 37). We observed that all three signaling pathways were activated after CPSIT_0556 stimulation of hPMN. However, the activation of intracellular signaling alone does not necessarily mean that the signaling is involved in the delayed apoptosis response. Although p38 MAPK phosphorylation increased in hPMN stimulated by CPSIT_0556, activation of p38 MAPK did not promote the survival of hPMN following infection, as our study showed that exposure to p38 MAPK inhibitors did not affect the delayed apoptosis of CPSIT 0556-infected hPMN.

Chlamydia has developed mechanisms to interfere with pro-apoptotic and anti-apoptotic signals to properly control the time of cell death to ensure their survival and reproduction in host cells (16). The main pathways involved in hPMN apoptosis are the intrinsic and extrinsic pathways, both of which involve activation of caspases as the endpoint. The extrinsic pathway is mainly mediated by Fas receptors, while the intrinsic pathways can be activated by a variety of factors, such as cellular stress, including growth factor deprivation or DNA damage (65). Similar to *C. pneumoniae*, CPSIT_0556 affects PMNs survival by inhibiting spontaneous PMNs caspase-3 activation, which is a key enzyme in the hydrolytic cleavage of many cell target proteins, ultimately leading to DNA fragmentation and cell death (**Figure 2C**). Interactions between pro-apoptotic and anti-apoptotic Bcl-2 family members control mitochondrial apoptosis pathways (66). Thus, we evaluated the equilibrium between proapoptotic and antiapoptotic signals in maintaining mitochondrial integrity in CPSIT_0556-stimulated hPMN. Mcl-1 belong to the anti-apoptotic B-cell lymphoma 2 (Bcl-2) family (67), and its binding to certain pro-apoptotic members of the Bcl-2 family, inhibits hPMN apoptosis (68). Bax and Bak have three homologous domains of Bcl-2, which are essential for increasing the permeability of mitochondrial membrane and the release of cytochrome C. Other pro-apoptotic proteins exhibit only the Bcl-2 homology 3 (BH3) domain. These “BH3-only” proteins bind and inhibit anti-apoptotic Bcl-2 family members, thereby releasing pro-apoptotic Bax and Bak proteins, which leads to the loss of mitochondrial membrane permeability and subsequent cell death (68, 69).

MnSOD has been identified as a mitochondrial marker of hPMN (53). Bax is mainly localized in the cytoplasm and redistributes to mitochondria under the stimulation of apoptosis where it forms aggregates (70, 71). However, it remains unclear so far which molecules are responsible for Bax translocation to the mitochondria in hPMN. It has been reported that the PI3K/Akt pathway regulates the translocation of Bax to the mitochondria in HeLa cells (71). Thus, we investigated whether CPSIT_0556 could inhibit the transport of Bax to the mitochondria and its effects on the PI3K/Akt pathway and Bax expression in hPMN. Western blotting showed that Bax waned, and Mcl-1 expression was significantly increased in hPMN exposed to CPSIT_0556, while treatment with the PI3K inhibitor down-regulated the expression of Mcl-1 and up-regulated the expression of Bax **(** **Figure 5A**). Furthermore, immunofluorescence results showed that Bax was uniformly distributed in the cytoplasm at 6 h, and STS promoted the co-localization of Bax and mitochondria in neutrophils. At 24 h, the hPMNs showed similar result as the STS-treated group, presenting the co-localization of Bax and mitochondria. CPSIT_0556 inhibited the translocation of Bax in the hPMN to the mitochondria, while inhibition of the PI3K/Akt pathway promoted the entry of Bax into the mitochondria, which indicated that CPSIT_0556 inhibited the translocation of Bax into mitochondria through the PI3K/Akt pathway (**Figure 5B**). During hPMN cell death, protein levels of Mcl-1 decreased gradually, leading to the release of Bax from Mcl-1 (26), we confirmed that CPSIT_0556 could up-regulate the expression of Mcl-1. Thus, CPSIT_0556 may inhibit the transfer of Bax from the cytoplasm to mitochondria by promoting the expression of Mcl-1.

IL-8 is not only an effective hPMN attractor and activator, but also plays an important role in acute lung injury (72), and is believed to be an amplification mechanism involved in recruiting large amounts of hPMN to sites of infection (73). *In vitro*, hPMN rarely secrete IL-8, however, upon stimulation, hPMN produce a range of cytokines, including G-CSF and GM-CSF (74), as well as IL-8 (75). In this study, CPSIT_0556 secretes IL-8 through PI3K/Akt and NF-κB pathways, and IL-8 enhances Akt phosphorylation through PI3K-dependent pathways. Since the PI3K/Akt pathway regulates the expression of Mcl-1, we explored the relationship between IL-8 and Mcl-1. The results showed that IL-8 up-regulated the expression of Mcl-1.

IL-8 has been reported to promote the survival of adjacent hPMN through a paracrine mechanism (40). We explored the regulatory effects of CPSIT_0556 on IL-8-release by hPMN on apoptosis. Anti-IL-8 mAb and protein A-agarose were used to deplete IL-8 from CPSIT_0556-hPMN supernatants, and then incubated hPMN for 24 h. Subsequently, we demonstrated that the apoptosis rate of the hPMNs treated with CPSIT_0556 cultured with supernatant decreased, the apoptosis rate hPMNs cultured in IL-8-depleted medium was higher than that of the hPMNs treated with CPSIT_0556 with un-depleted IL-8 supernatant, while the recombinant human IL-8 had no effect on the apoptosis of hPMN. Interestingly, Western blotting results also showed that recombinant human IL-8 at different concentrations could not down-regulate the expression of Bax (**Figure 7D**). IL-8 anti-apoptotic effect may require cooperation with other cytokines, which needs further study.

## Conclusion

Intracellular bacteria have evolved a variety of mechanisms to enhance their survival and replication in host cells (76), but the role of Incs in Chlamydia immune escape *via* hPMN killing remains poorly understood. This study provides new insight into the biological functions of CPSIT_0556, and demonstrated that CPSIT_0556 inhibited hPMN apoptosis through PI3K/Akt and NF-κB pathways, released IL-8, inhibited the activation of procaspase-3, up-regulated the expression of Mcl-1, down-regulated the expression of Bax, and inhibited the translocalization of Bax from the cytoplasm to mitochondria, and induced p65 NF-κB transfer from the cytoplasm to the nucleus, which may be related to the intrinsic pathway of apoptosis. Further research is needed to identify the receptor that mediates the binding of CPSIT_0556 to hPMN, and to explore the potential application of CPSIT_0556 as a vaccine or therapeutic target for the prevention and treatment of psittacosis.

## Data Availability StateMent

The datasets presented in this study can be found in online repositories. The names of the repository/repositories and accession number(s) can be found in the article/supplementary material.

## Ethics Statement

The studies involving human participants were reviewed and approved by Ethics Committee of the University of South China and is in accordance with Declaration of Helsinki. The patients/participants provided their written informed consent to participate in this study. The animal study was reviewed and approved by Ethics Committee of the University of South China.

## Author ContribuTions

ZH, KZ, and SL contributed to conception and design of the study. The experiment was completed by ZH, JX, MY, and JW. ML analyzed experimental results and data. CW and ND guided the design of the study and revised the manuscript. All authors contributed to the article and approved the submitted version.

## Funding

This project was supported by the National Natural Science Foundation of China (31872643 , 31800162), Natural Science Foundation of Hunan Province (No. 2020JJ5444), the Hunan Provincial Key Laboratory for Special Pathogens Prevention and Control Foundation under Grant No. 2014-5, and the Hunan Province Cooperative Innovation Center for Molecular Target New Drug Study (2015-351).

## Conflict of Interest

The authors declare that the research was conducted in the absence of any commercial or financial relationships that could be construed as a potential conflict of interest.

## Publisher’s Note

All claims expressed in this article are solely those of the authors and do not necessarily represent those of their affiliated organizations, or those of the publisher, the editors and the reviewers. Any product that may be evaluated in this article, or claim that may be made by its manufacturer, is not guaranteed or endorsed by the publisher.

## Abbreviations

Inc, Inclusion membrane proteins; hPMN, Human polymorphonuclear neutrophils; STS, staurosporine.
